# Linking knowledge, belief and awareness of menstrual cycle with oral health in young India women

**DOI:** 10.6026/973206300222993

**Published:** 2026-05-31

**Authors:** Subhash Kumar, Qalbi Fatima, Pallavi Priya, Imran Md, Vaibhava Raaj, Abhishek Sinha

**Affiliations:** 1 Department of Dentistry, Patna Medical College & Hospital, Patna, Bihar, India

**Keywords:** Menstrual cycle, oral health, knowledge, awareness, gingival health, young women

## Abstract

Limited awareness of the relationship between menstrual cycle-related hormonal changes and oral health remains a concern among young women, particularly in developing settings. Hence, this study assessed knowledge, beliefs and awareness regarding menstrual cycle and its correlation with oral health among women aged 18-30 years attending a government medical college. A total of 400 participants completed a validated questionnaire and underwent clinical examination using Gingival Index and Oral Hygiene Index-Simplified. Although general menstrual knowledge was adequate in 67.5% of participants, only 18.8% demonstrated awareness of its impact on oral health, with higher gingival inflammation observed in those with poor awareness and irregular cycles. Significant knowledge gaps highlight the need for targeted educational interventions integrating menstrual and oral health promotion.

## Background:

Menstruation is a natural physiological process in women of actual reproductive ability, wherein cyclical hormonal changes that involve majorly estrogen and progesterone hormones are the ones that dictate the monthly preparation and shedding of the endometrial lining [1]. In addition to reproductive implications, the menstrual cycle has systemic biological effects, which are found in various organ systems such as the oral cavity and sex steroid hormone receptors are found in the gingival tissue, salivary glands, temporomandibular joint structures and oral mucosal epithelium [2]. These hormonal effects cause the oral cavity to be a hormonally sensitive space that experiences periodical changes in tandem with the menstrual cycle, which has become more and more established in the literature of periodontal and oral medicine during the past few decades [3]. The effect of sex hormones on periodontal tissues is well established at the molecular condition. Estrogen and progesterone adjust the gingivascular activity by raising capillary permeability and vasodilation, changing the composition and metabolic activity of the periodontopathogenic microbial flora such as Prevotella intermedia, inhibiting the local immune response by decreasing polymorphonuclear leukocyte chemotaxis and affecting collagen synthesis and breakdown in the gingivocalicular interstitium [4]. The clinical event of menstruation-related gingivitis is the result of these hormonal effects, where the level of gingival bleeding, edema and erythema peaks during the premenstrual and menstrual cycle and decreases during the postmenstrual phase [5]. The affected oral changes caused by hormones during the menstrual period can manifest as bleeding of the gums during brushing or flossing, oral ulceration especially aphthous lesions, high viscosity of salivary, distortion of taste perception, temporomandibular pain and increase in oral mucosa sensitivity [6]. Even though these symptoms are usually self-limiting and temporary, they may have a strong effect on oral discomfort, diet and oral health compliance at the time of the affected periods of the cycle. Moreover, the sustained effects of regular hormonal changes every month over years of reproductive level can also play a part in the progressive development of periodontal disease in those who are predisposed to it, especially those who already have gingivitis or periodontitis [7]. Although the relationship between menstrual hormones and oral reactions has been well established biologically, the general population of women has very little awareness about such relation. Research has been carried out across different nations has continuously shown that most women do not know their menstrual cycle can influence their oral health and therefore they do not report their menstrual based oral symptoms to their dental specialists or alter their oral hygiene routines during hormonally active times [8]. This knowledge gap is especially high among developing nations where even the fact of menstruation is still enveloped by cultural taboos, misconceptions and social stigma that prevents the open discussion, health education and a behavioral inclination to seek health care with regard to menstrual health [9]. In India, the topic of menstruation is still rather socio-culturally sensitive and the traditional beliefs and practices associated with it remain strong and regional, religious and socio-economic divisions. It has been reported in numerous studies that Indian women and adolescent girls have huge misunderstandings about menstruation and many of them believe it to be impure, shameful or diseased instead of a normal physiologic procedure [10]. Such beliefs shape menstrual hygiene practices, attitudes to healthcare seeking behavior and attitudes about reproductive health and can be generalized to include perceptions about systemic health effects of menstruation and its oral health effects [11]. The city of Bihar and the state, in particular, has been especially well placed to explore the subject of menstrual health literacy because of its unique sociodemographic traits such as reduced female literacy, restricted access to reproductive health education in rural feeder zones and the presence of traditional menstrual beliefs and taboos that have been recorded in surveys conducted in the state [12]. The young females attending a government medical college dental department are a population that is actively working with the healthcare system but their knowledge of the hormonal-oral health relationship cannot be deemed to be sufficient. The existing literature on menstrual knowledge and its connection with oral health has largely been carried out in a population of the West or in an urban metropolitan region within India and the results might not be easily applicable to the population with other sociocultural backgrounds, a different education level and oral health care access patterns [13]. Moreover, the available literature has also mainly evaluated either menstrual knowledge or oral health status without directly exploring both and how they relate to each other among the same sample of study participants. There have been a few studies that have examined the relationship between the presence of particular knowledge gaps, cultural beliefs and quantifiable clinical oral health parameters in a systematic manner [14]. Thus, the purpose of the current research was to evaluate the knowledge, beliefs and awareness of menstrual cycle and its relation to oral health among young women who attend the Department of Dentistry at Government Medical College, Patna and to determine the association between the cognitive variables and clinical oral health variables. The null hypothesis was that there would be no remarkable relationship between the menstrual-oral health awareness levels and the clinical gingivital health conditions among the study participants. Therefore, it is of interest to evaluate the knowledge, beliefs and awareness of menstrual cycle and its association with oral health among young women.

## Materials and Methods:

## Study design and setting:

This cross-sectional observational study was conducted at the Department of Dentistry, Government Medical College, Patna, Bihar, India, from December 2025 to March 2026. Written informed consent was obtained from all participants after detailed explanation of the study objectives and procedures in both English and Hindi.

## Sample size calculation:

The sample size was calculated using the formula for cross-sectional studies: n = Z^2^pq/d^2^, where Z = 1.96 (95% confidence level), p = estimated proportion of women with adequate menstrual-oral health awareness (assumed 20% based on previous literature), q = 1 - p and d = margin of error (4%). The calculated minimum sample size was 385 participants. To account for incomplete responses and potential exclusions, 420 women were initially approached and 400 who met all criteria and provided complete responses were included in the final analysis.

## Participant selection:

Young women aged 18 to 30 years attending the dental outpatient department for routine dental consultation or treatment were consecutively recruited during the study period.

## Inclusion criteria:

[1] Age between 18 and 30 years

[2] Regular menstrual cycles established for a minimum of two years

[3] Willingness to participate and provide informed consent

[4] Ability to read and comprehend Hindi or English

## Exclusion criteria:

[1] Pregnant or lactating women

[2] Women using hormonal contraceptives within the preceding six months

[3] History of polycystic ovarian syndrome or other diagnosed endocrine disorders

[4] Current use of medications known to affect gingival tissues (phenytoin, cyclosporine, calcium channel blockers)

[5] History of periodontal treatment within the preceding three months

[6] Women with fewer than 20 natural teeth

[7] History of hysterectomy or surgical menopause

[8] Diagnosed psychiatric conditions affecting comprehension or consent

## Questionnaire development and validation:

A structured, self-administered questionnaire was developed specifically for this study based on an extensive review of existing menstrual health literacy instruments and oral health knowledge assessment tools. The questionnaire was designed in English, translated into Hindi by a certified bilingual translator and back-translated to verify conceptual equivalence. Content validity was established through review by a panel of five experts comprising two periodontists, one gynecologist, one public health specialist and one health education expert. Each item was rated for relevance; clarity and appropriateness on a four-point scale and items achieving a content validity index below 0.80 were revised or eliminated. The questionnaire was pilot tested on 40 women (not included in the final study) to assess comprehension, completion time and internal consistency. The pilot testing yielded a Cronbach's alpha of 0.83, indicating good internal reliability. Average completion time was 15 to 20 minutes.

## The final questionnaire comprised four sections:

[1] Section A - Demographic Information (8 items): Age, education level, marital status, occupation, monthly family income, residential background (urban/rural), source of menstrual health information and regularity of dental visits.

[2] Section B - Menstrual Knowledge (12 items): Questions assessing understanding of menstrual physiology, cycle duration, hormonal basis, menstrual hygiene practices and recognition of normal versus abnormal menstruation. Each correct response received one point (maximum score: 12). Scores were categorized as: adequate knowledge (≥ 9), moderate knowledge (5-8) and poor knowledge (≤ 4).

[3] Section C - Menstrual Beliefs and Cultural Perceptions (10 items): Items assessing traditional beliefs, taboos and cultural practices related to menstruation, including restrictions on daily activities, food, religious practices and social interactions. Responses were recorded on a five-point Likert scales ranging from strongly agree to strongly disagree. Higher scores indicated more progressive attitudes with fewer restrictive beliefs.

[4] Section D - Awareness of Menstrual-Oral Health Connection (10 items): Questions evaluating awareness of hormonal effects on oral tissues, menstruation-associated gingival changes, the importance of modified oral hygiene during menstruation and recognition of oral symptoms related to the menstrual cycle. Each correct response received one point (maximum score: 10). Scores were categorized as: adequate awareness (≥ 7), moderate awareness (4-6) and poor awareness (≤ 3). Additionally, a supplementary section captured self-reported oral health experiences during menstruation, including questions about gingival bleeding, mouth sores, oral pain, dry mouth and changes in oral hygiene behavior during the premenstrual or menstrual phase.

## Clinical oral examination:

Following questionnaire completion, a standardized clinical oral examination was performed by a single calibrated examiner under artificial illumination using a dental mirror, periodontal probe (UNC-15) and explorer. The following clinical indices were recorded:

[1] Gingival index (GI) - Löe and Silness (1963): Assessed on six index teeth (16, 12, 24, 36, 32, 44) at four sites per tooth (mesial, distal, buccal, lingual). Scores: 0 = normal gingiva, 1 = mild inflammation, 2 = moderate inflammation, 3 = severe inflammation.

[2] Oral hygiene index-simplified (OHI-S) - Greene and Vermillion (1964): Comprising the Debris Index (DI-S) and Calculus Index (CI-S), assessed on six index surfaces. OHI-S scores were categorized as: good (0-1.2), fair (1.3-3.0) and poor (3.1-6.0). To control for the hormonal influence on gingival assessment timing, clinical examinations were performed during the follicular phase of the menstrual cycle (days 5-12) whenever scheduling permitted. For participants examined during other phases, the menstrual phase at the time of examination was recorded as a covariate.

Intra-examiner reliability was assessed by duplicate examination of 30 randomly selected participants at a one-week interval, yielding an intraclass correlation coefficient of 0.91 for GI and 0.93 for OHI-S.

## Statistical analysis:

Data were entered into Microsoft Excel and analyzed using SPSS version 25.0 (IBM Corp., Armonk, NY, USA). Descriptive statistics were presented as mean ± standard deviation for continuous variables and frequencies with percentages for categorical variables. Chi-square tests were used for associations between categorical variables. Independent samples t-tests and one-way ANOVA with post-hoc Tukey's test were used for comparing means across groups. Spearman's rank correlation was used to assess relationships between knowledge/awareness scores and clinical indices. Multiple linear regression analysis was performed with GI as the dependent variable and awareness score, OHI-S, age, education level and menstrual regularity as independent variables. Statistical significance was set at p < 0.05.

## Results:

The 400 participants had a mean age of 23.6 ± 3.4 years. The demographic profile and menstrual knowledge scores are presented in Table 1. Cultural beliefs, menstrual-oral health awareness levels and self-reported oral symptoms during menstruation are presented in Table 2. The majority of participants held at least one restrictive cultural belief regarding menstruation. Awareness of the menstrual-oral health connection was markedly deficient, with only 18.8% demonstrating adequate awareness. A substantial proportion of participants reported experiencing oral symptoms during menstruation but did not attribute them to hormonal changes. Clinical oral health parameters stratified by awareness level and correlation analysis results are presented in Table 3. Participants with poor menstrual-oral health awareness demonstrated significantly higher Gingival Index scores and poorer oral hygiene compared to those with adequate awareness. Significant negative correlations were observed between awareness scores and GI values. Post-hoc Tukey's test revealed significant differences in GI scores between all three awareness level groups (poor vs. moderate: p < 0.001; poor vs. adequate: p < 0.001; moderate vs. adequate: p = 0.003). Women with irregular menstrual cycles (n = 76, 19.0%) demonstrated significantly higher mean GI scores (1.58 ± 0.51) compared to women with regular cycles (1.24 ± 0.46, p < 0.001). Women from rural backgrounds had significantly lower menstrual- oral health awareness scores (3.48 ± 2.21) compared to urban women (4.59 ± 2.54, p < 0.001).

## Discussion:

The current research fully evaluated the knowledge, perceptions and sensitization about menstrual cycle and its connection with the oral health in young women visiting a government dental clinic at Patna and determined that there were strong connections among these mental constructs and clinical oral health variables. The results showed that there is a dramatic difference between sufficient general knowledge of menstruation (67.5) and significantly poor knowledge of the menstrual-oral health relationship (18.8) and thus it is found that basic menstrual literacy has been enhanced among the young Indian women, but there is still no integration of oral health views in menstrual health education. The fact that the proportion of those people who had heard about hormonal changes having the impact on gingival health is only 28.5% and that only 11.3% of the participants had heard of the concept of menstrual gingivitis, demonstrates a serious deficit in education with immediate clinical consequences. Hormonal changes in the gingivality tissues during the menstrual cycle are not a new inding and the historic researches have shown that the flow of gingivality crevicular fluid, the levels of gingivality bleeding and the levels of subgingival colonization by gram-negative anaerobes are higher in ovulatory and premenstrual periods [15]. However, this scientific body of knowledge has evidently not been converted into the communicative of health to the population or internalized into the menstrual health education that young women are exposed to through their main points of generation. The domination of mothers and family members as the main source of menstrual information (54.0%) is in line with the trends recorded in South Asian countries whereby the intergenerational transmission of menstrual knowledge is practiced largely in the intimate family setting and not in education sectors [16]. The given pathway of transmission through the family is culturally suitable, but it is prone to the spread of the traditional beliefs and practices along with the facts and is necessarily restricted by the knowledge base of the informing family member. The poor role of formal education (14.0%) as a source of menstrual information is alarming and indicative of the failure of menstrual health education in the school education system in most of the Indian states [17]. The high correlation between clinical gingivality and menstrual-oral health awareness is worth interpreting. The correlation between scores on awareness and Gingival Index value (r = -0.48) and the substantial independent effect of awareness to the regression model indicates that the women who realize the effect that hormones have on their gums tend to have better gums. This association is probably mediated via behavioral mechanisms, i.e. women who know that their gums might be more susceptible at some time of their menstrual cycle are presumably more motivated to adhere to regular oral hygiene behaviors and are less likely to be motivated to cut back on brushing behavior during menstruation [18]. The conclusion that is raised by the fact that 27.0% of the participants stated that they brush their teeth less frequently during their menstrual period is clinically relevant and could indicate the overlap of physical discomfort with the cultural limitations and lack of knowledge. Other women can lower oral care because of menstruation in general malaise, abdominal pain, tiredness, which decrease the motivation to do self-care activities. Others can do it because of the cultural beliefs that restrict diverse physical activities during the menstrual month. Moreover, women that have a greater loss of gingival blood during brushing can misleadingly conclude this as a message to brush less not as an indication of periodontal inflammation needing increased processing [19]. The shown behavioral pattern is unproductive as the premenstrual and menstrual phases are the times when the gingival vulnerability is the highest and the time when the plaque removal is the most crucial task. The continuity of the restrictive cultural beliefs on menstruation among such a large percentage of the participants (46.0% regarding menstruation as impure and 67.0% regarding menstrual avoidance of religious settings) is also consistent with the results of various studies carried out in India that have recorded the effects of traditional menstrual taboos at educational and socioeconomic levels [20]. The positive correlation between restrictive beliefs and gingival inflammation (r = 0.29) is significant and could be attributed to the wider implication of health-seeking behavior of the restrictive beliefs: women with more restrictive views about menstruation might not be as willing to discuss menstrual-related health issues with healthcare providers, may be less responsive to health education messages about menstruation, or be more likely to accept menstrual discomfort and other associated symptoms including oral symptoms [21]. The observed high GI scores of women with irregular menstrual cycles than regular ones are biologically reasonable. Incorrect cycles usually manifest the hormonal dysfunction, such as subclinical polycystic ovarian syndrome, hypothalamic malfunction, or thyroid disease, which can result in abnormal or exaggerated changes in hormonal levels of the inflammatory response in gingival tissues [22]. The findings support the significance of asking about the regularity of menstruation in completing dental history taking, as it can act as a clinical marker of increased periodontal vulnerability. The urban rural difference in the score of menstrual- oral health awareness is in line with the wider trends of the health literacy gap in the Indian setting. The rural issues are characterized by low access to resources of health education, less contact with the medical personnel and more robust traditional beliefs, factors leading to a low level of awareness of the systemic effects of menstruation [23]. This gap indicates the need to have geographically focused learning interventions and one area that is in need is rural and semi-urban Bihar. The fact that the percentage of participants who had ever talked to a dentist about menstrual-related oral symptoms is extremely low (6.5 percent) displays a two- way communication gap. Patients can also fail to provide this information out of embarrassment, ignorance of the relevance of this information, or culture-based taboos regarding discussing menstruation with dentists, most of whom are male in the region. On the other hand, dentists might not regularly ask questions about how menstrual health is when it comes to evaluating patients, which is a more general sign of a lack of incorporation of systemic health into dental practice [24]. There are various limitations that should be mentioned. The cross-sectional type restricts the ability to be causal in the direction of the association between the awareness and gingival health. The research sample was selected on the basis of one institutional environment and therefore it might not be sufficient to generalize to the wider population of Bihar or other states in India. Self-reports on oral symptoms during menstruation are prone to recall bias and may not necessarily represent the temporal association between menstrual cycle and oral manifestations. The clinical assessment was conducted at one point and in an ideal scenario the longitudinal evaluation of changes in gingiva during several menstrual cycles would be a stronger evidence of cyclical changes in the gingiva [25]. Although they tried to arrange examination around the follicular follicle, not all of the participants could be examined around the follicle and that may have caused hormonal confounding of the gingivism examination.

## Conclusion:

Young India women demonstrated adequate general knowledge of menstruation but poor awareness of its impact on oral health, highlighting a significant knowledge gap. Reduced awareness was associated with poorer gingival health and underreporting of menstrual-related oral symptoms. Integrating menstrual health education with oral health promotion may improve clinical outcomes and patient awareness.

## Figures and Tables

**Table 1:** Demographic characteristics and menstrual knowledge levels (n = 400)

**Characteristic**	**n (%) or Mean ± SD**
**Age Group**	
18-22 years	156 (39.0%)
23-26 years	148 (37.0%)
27-30 years	96 (24.0%)
**Education Level**	
Up to secondary (10th)	84 (21.0%)
Higher secondary (12th)	112 (28.0%)
Graduate and above	204 (51.0%)
**Residential Background**	
Urban	228 (57.0%)
Rural	172 (43.0%)
**Marital Status**	
Unmarried	248 (62.0%)
Married	152 (38.0%)
Regular Dental Visits (≥ 1/year)	92 (23.0%)
**Source of Menstrual Information**	
Mother/family	216 (54.0%)
Friends/peers	88 (22.0%)
School/teacher	56 (14.0%)
Media/internet	40 (10.0%)
**Menstrual Knowledge Level**	
Adequate (≥ 9)	270 (67.5%)
Moderate (5-8)	98 (24.5%)
Poor (≤ 4)	32 (8.0%)
Menstrual Knowledge Score	8.42 ± 2.31

**Table 2:** Menstrual beliefs, oral health awareness and self-reported oral symptoms

**Parameter**	**n (%)**
**Prevalent Cultural Beliefs (Agreement)**	
Menstruation is impure/unclean	184 (46.0%)
Should not visit religious places during menses	268 (67.0%)
Dietary restrictions necessary during menses	152 (38.0%)
Should avoid physical exercise during menses	196 (49.0%)
Should not discuss menstruation openly	224 (56.0%)
**Menstrual-Oral Health Awareness Level**	
Adequate (≥ 7)	75 (18.8%)
Moderate (4-6)	137 (34.2%)
Poor (≤ 3)	188 (47.0%)
Awareness Score, Mean ± SD	4.12 ± 2.47
**Specific Awareness Items (% Correct)**	
Aware that hormones affect gums	28.50%
Aware that gums may bleed more during menses	34.00%
Aware that oral hygiene should be maintained during menses	62.50%
Aware of menstrual gingivitis concept	11.30%
Ever discussed menstrual oral symptoms with dentist	6.50%
Self-Reported Oral Symptoms During Menstruation	
Gingival bleeding during brushing	176 (44.0%)
Mouth ulcers/sores	128 (32.0%)
Oral pain or discomfort	96 (24.0%)
Dry mouth sensation	72 (18.0%)
Altered taste	56 (14.0%)
Reduced brushing frequency during menses	108(27.0%)

**Table 3:** Clinical oral health parameters by awareness level and correlation analysis

**Panel A: Clinical Parameters by Awareness Level**	**Poor Awareness (n=188)**	**Moderate Awareness (n=137)**	**Adequate Awareness (n=75)**	**p-value(ANOVA)**
Gingival Index, Mean ± SD	1.54 ± 0.48	1.21 ± 0.42	0.87 ± 0.36	< 0.001
OHI-S Score, Mean ± SD	2.68 ± 0.94	2.14 ± 0.82	1.52 ± 0.71	< 0.001
DI-S Score, Mean ± SD	1.62 ± 0.58	1.28 ± 0.51	0.94 ± 0.43	< 0.001
CI-S Score, Mean ± SD	1.06 ± 0.52	0.86 ± 0.44	0.58 ± 0.38	< 0.001
GI Category - Moderate/Severe, n (%)	134 (71.3%)	62 (45.3%)	18 (24.0%)	< 0.001
**Panel B: Spearman Correlations with GI**	**r**	**p-value**		
Menstrual-oral health awareness score	-0.48	< 0.001		
Menstrual knowledge score	-0.31	< 0.001		
Cultural belief score (restrictive)	0.29	< 0.001		
OHI-S score	0.62	< 0.001		
Education level	-0.36	< 0.001		
Regular dental visits	-0.27	< 0.001		
**Panel C: Multiple Regression-Dependent Variable: GI**	**β**	**p-value**		
OHI-S score	0.41	< 0.001		
Menstrual-oral health awareness score	-0.24	< 0.001		
Education level	-0.13	0.012		
Menstrual regularity (irregular = 1)	0.11	0.034		
Age	0.08	0.127		
R^2^=0.52,F = 84.67, p < 0.001				
